# CscoreTool-M infers 3D sub-compartment probabilities within cell population

**DOI:** 10.1093/bioinformatics/btad314

**Published:** 2023-05-11

**Authors:** Xiaobin Zheng, Joseph R Tran, Yixian Zheng

**Affiliations:** Department of Embryology, Carnegie Institution for Science, Baltimore, MD 21218, United States; Department of Embryology, Carnegie Institution for Science, Baltimore, MD 21218, United States; Department of Embryology, Carnegie Institution for Science, Baltimore, MD 21218, United States

## Abstract

**Motivation:**

Computational inference of genome organization based on Hi-C sequencing has greatly aided the understanding of chromatin and nuclear organization in three dimensions (3D). However, existing computational methods fail to address the cell population heterogeneity. Here we describe a probabilistic-modeling-based method called CscoreTool-M that infers multiple 3D genome sub-compartments from Hi-C data.

**Results:**

The compartment scores inferred using CscoreTool-M represents the probability of a genomic region locating in a specific sub-compartment. Compared to published methods, CscoreTool-M is more accurate in inferring sub-compartments corresponding to both active and repressed chromatin. The compartment scores calculated by CscoreTool-M also help to quantify the levels of heterogeneity in sub-compartment localization within cell populations. By comparing proliferating cells and terminally differentiated non-proliferating cells, we show that the proliferating cells have higher genome organization heterogeneity, which is likely caused by cells at different cell-cycle stages. By analyzing 10 sub-compartments, we found a sub-compartment containing chromatin potentially related to the early-G1 chromatin regions proximal to the nuclear lamina in HCT116 cells, suggesting the method can deconvolve cell cycle stage-specific genome organization among asynchronously dividing cells. Finally, we show that CscoreTool-M can identify sub-compartments that contain genes enriched in housekeeping or cell-type-specific functions.

**Availability and implementation:**

https://github.com/scoutzxb/CscoreTool-M.

## 1 Introduction

One important question in biology pertains to how DNA is packaged and organized in the small nuclear space of eukaryotes so that gene expression can be temporally and spatially regulated during organism development and homeostasis. The nucleus is a complicated structure with many functional subunits, such as the nuclear speckles, the nucleolus, and the nuclear lamina. These units have been recognized as organization hubs of the genome for specific functions. How these hubs are organized with respect to one another and to the 3D chromatin interactions remains, however, poorly understood. As a result, it is unclear how the different genomic units work together to regulate gene expression in the context of organism development and function.

The genomic sequences associated with the nuclear lamina, speckles, and nucleoli have been mapped (Guelen et al. 2008; Németh et al. 2010; Chen et al. 2018). The genes in the nuclear lamina-associated chromatin domains (LADs) are largely silenced and the LADs are generally gene-poor with overall repressive epigenetic features. Nuclear speckles are enriched for transcribed genes that create a transcriptionally active environment. Thus, chromatin associated with the speckles show active epigenetic features. It remains unclear if the actively transcribed house-keeping genes and cell-type specific genes are organized into the same or different speckles and if the organization shows any cell type specificity. Nucleoli are involved in transcribing ribosomal RNAs and ribosome assembly. Although nucleoli are mostly found away from the nuclear periphery, the nucleolus-associated chromatin domains (NADs) largely overlap with LADs. In addition, lamins, the major structural component of the nuclear lamina, have been found to be associated with nucleolus (Martin et al. 2009; Sen Gupta 2017), but it is unclear whether this can fully explain the overlap observed between NADs and LADs. Moreover, how NADs and LADs are organized with respect to one another and to the rest of the genome is poorly understood.

Many efforts have been devoted to understanding nuclear and genome organization. Among the numerous methods developed, the imaging approaches, such as fluorescence in situ hybridization to visualize DNA, RNA, and immunostaining to visualize proteins, allow simultaneous visualization of proteins, DNA, and RNA transcripts in individual cells. Whereas imaging approaches are important to understand the heterogeneity of nuclear and genome organization and transcriptional activity in cells, they are limited to regions of the nucleus where the probes are designed. Recent advances in barcoding and iterative hybridization approaches have enabled imaging of thousands of genomic loci in the same cell (Bintu et al. 2018; Takei et al. 2021), but the resolution is still limited.

To overcome the limitations of imaging approaches, direct DNA sequencing has been used to study genomic regions associated with specific nuclear substructures, transcription factors, or epigenetic modifications. The *Ch*romatin *I*mmuno*P*recipitation followed by massive parallel *seq*uencing (ChIP-seq) (Mikkelsen et al. 2007) has been used extensively to understand epigenetic modification and transcription factor interaction with chromatin, but the method does not offer information on 3D chromatin organization or sub-nuclear structures. Several methods, including DNA adenine methylase identification (DamID) (Guelen et al. 2008), nucleolar sequencing (Németh et al. 2010), Ascorbate Peroxidase (APEX)-based map of LADs (Tran et al. 2021), Tyramide-Signal Amplification (TSA)-seq (Chen et al. 2018), and chromatin pull down based TSA-seq (cTSA-seq) (Tran et al. 2022) have been developed to map the genomic regions associated with nuclear lamina, nucleolar, and nuclear speckles. Finally, chromatin conformation capture based methods, including Hi-C (Lieberman-Aiden et al. 2009), were developed to identify interactions between different chromatin regions, which allowed the reconstruction of the 3D chromatin interaction and folding. However, more computational efforts are still needed to fill in the gaps between the DNA-DNA interaction profile and nuclear substructure-associated chromatin domains.

The A/B compartment concept was introduced in the first Hi-C study. By applying principal component analysis to the distance-normalized Hi-C interaction profile, Lieberman-Aiden et al. discovered that the genome can be separated into the two large compartments, namely the A- and B-compartment (Lieberman-Aiden et al. 2009). Genomic regions in the A-compartment have a higher chance to interact within the A-compartment, while genomic regions in the B-compartment have a higher chance of interactions with one another. By analyzing epigenome, transcriptome, and LADs in A/B compartments, it became clear that B compartments contain LADs and additional heterochromatin regions, while the A compartments were enriched for active euchromatin. We developed a model-based method, CscoreTool, to provide A–B compartment inference at higher resolution, better accuracy, and lower computational cost (Zheng and Zheng 2018; Zheng et al. 2018). These compartment studies suggest that the 3D chromatin interactions mapped by Hi-C can be used to define local chromatin compartments that share high interactions within each compartment.

To reveal more detailed 3D chromatin compartments, Rao et al. used high-resolution Hi-C and Gaussian Hidden Markov Model-based clustering method to generate six sub-compartments and their further analyses linked some of these sub-compartments to the LADs, NADs, and speckle-associated chromatin (Rao et al. 2014). Although the algorithm used by Rao et al. represents an important progress in identifying the sub-compartments of the genome, there are several limitations. For example, the method arbitrarily separates the genome into “even” and “odd” chromosomes and clusters them separately. The compartments inferred from the even and odd chromosomes may not match each other. In addition, the method does not take into consideration the heterogeneity of genome interactions in individual cells known to exist even within a given cell type. Another recent method, called SNIPER (Xiong and Ma 2019), used a neural network to “impute” low-depth dataset and inferred sub-compartments. However, this method is supervised and depends on Rao et al.’s results using the GM12878 dataset. Thus, SNIPER suffers the same limitations, and it could not be used to infer more than six sub-compartments or be applied to other organisms without a training dataset. Recently, Calder (Liu et al. 2021) and SCI (Ashoor et al. 2020) were developed as new unsupervised methods to infer sub-compartments from bulk Hi-C data, but these two methods still do not consider the heterogeneity with cell population.

Here, we introduce CscoreTool-M, a model-based tool to infer multiple sub-compartments of the genome. CscoreTool-M extends the model used in CscoreTool to accommodate more sub-compartments. The compartment scores (Cscores) provided by this method are equal to the probability (or percentage) of cells within the population that a genomic region is in certain sub-compartments. We show that CscoreTool-M can reveal the heterogeneity of genomic sub-compartment relations within the cell population, and it offers a great opportunity to define finer sub-compartment organizations of the genome that are directly related to nuclear sub structures and their functions.

## 2 Materials and methods

### 2.1 Modeling sub-compartments based on Hi-C dataset obtained from cell populations

#### 2.1.1. Modeling sub-compartments in population Hi-C data

We assume that there are totally *M* sub-compartments in the nucleus, and the cell population can be heterogeneous, with each genomic region *i* located to sub-compartment *k* at a probability $P_{ik}$; k=1,…,M; $\sum_{k}P_{ik}=1$.

Like Rao et al. our model is based on *trans*-interactions (inter-chromosome interactions). Compared to *cis*-interactions (intra-chromosome interactions), *trans*-interactions are not affected by topological domains or other local constraints. Thus, the interaction frequencies are mainly determined by compartment structure.

The model is based on three hypotheses:

Hypothesis 1: For two different genomic loci, their nuclear sub-compartment locations in cells are independent.

Hypothesis 2: Two genomic loci located at different nuclear sub-compartments in one cell do not have 3D interaction in that cell.

Hypothesis 3: Two genomic loci located at the same sub-compartment in one cell have a constant probability of interaction.

Since we only use trans-interactions in the analysis, local structures such as TADs would not violate our hypotheses. However, one potential violation of hypothesis 1 is the Rabl effect in the nucleus. The Rabl effect shows centromere-centromere or telomere-telomere interactions among different chromosomes (Imakaev et al. 2012; Nagano et al. 2017; Stevens et al. 2017). These interactions would violate hypothesis 1 and make the *trans*-interactions at the centromeres or telomeres correlated. To account for the Rabl effect, we added a modification in our model. Assume the Rabl effect can be modeled by a term $R_{ij}$, then based on hypothesis 1, the probability that two genomic loci *i* and *j* are in the same compartment in a cell is:


(1) $$f_{ij}=R_{ij}\sum_{k}P_{ik}P_{jk}$$

Besides the Rabl effect, these three hypotheses may still be violated under various other conditions, and we will discuss the conditions when they are violated and the consequences in Section 2.2.

Based on hypothesis 2 and 3, the total number of interactions between two genomic loci should be in proportion to the probability that they are in the same genomic sub-compartment. Hi-C experiments are further affected by chromatin accessibility, genome mappability, ligation, and PCR efficiency. All these complication factors are summarized as a bias factor *b_i_* for each genomic locus (Imakaev et al. 2012), and the total interacting fragments in the Hi-C library could be modeled as:


(2) $$F_{ij}=B_{i}B_{j}f_{ij}=B_{i}B_{j}R_{ij}\sum_{k}P_{ik}P_{jk}$$

We further let $C_{ik}=B_{i}P_{ik}$, and the equation becomes:


(3). $$F_{ij}=R_{ij}\sum_{k}C_{ik}C_{jk}$$

The observed Hi-C interaction reads between genomic loci *i* and *j*, $N_{ij}$, can be modeled as a Poisson distribution with parameter $F_{ij}$, so we get the final likelihood function:


(4) $$L=\sum_{ij}\left[N_{ij}\ln\left(R_{ij}\sum_{k}C_{ik}C_{jk}\right)-R_{ij}\sum_{k}C_{ik}C_{jk}\right]$$

We further model the Rabl effect by a simple two-parameter function:


(5) $$R_{ij}=1+\alpha r_{i}r_{j}+\beta (r_{i}+r_{j})r_{i}r_{j}$$

Here, *r_i_* corresponds to the relative location of genomic locus *i* on the chromosome. It ranges from –0.5 (centromere) to 0.5 (telomere). α is the second-order coefficient modeling how strong the Rabl effect is, while β is the third-order coefficient modeling the skew (whether the centromere or telomere side has stronger interaction). This model is a simplified version of the third-order power series of of *R_ij_* as a function of *r_i_* (*x*) and *r_j_* (*y*):


$$f\left(x,y\right)=1+a_{10}x+a_{01}y+a_{20}x^{2}+a_{11}xy+a_{02}y^{2}+a_{30}x^{3}+a_{21}x^{2}y+a_{12}xy^{2}+a_{03}y^{3}+\cdots$$

We then removed all the items that had only *x* or *y* because those are not “interacting” items and the effects of these single-variable items can be reflected in the “bias factor”. This left only one second-order item: *xy*, and two third-order items: *x^2^y* and *xy^2^*. The coefficients of *x^2^y* and *xy^2^* are the same because the function should be symmetric. Finally, we got Equation (5) as a parametric form of the Rabl effect.

We can infer α, β, and *C_ik_* by maximizing the likelihood function in Equation (4) with constraints $C_{ik}\geq 0$ for all *i* and *k*, and then *B_i_* and *P_ik_* can be solved by and


(6) $$B_{i}=\sum_{k}C_{ik}$$

(7). $$P_{ik}=C_{ik}/B_{i}$$

#### 2.1.2. Optimization algorithm

The original optimization problem is non-convex and difficult to solve. However, note that when α, β, and all the other *C_jk_* are all fixed, the part of Equation (4), i.e. relevant to ***C***_*i*_ can be written as:


(8) $$L_{i}=\sum_{j}\left[N_{ij}\ln\left(\sum_{k}C_{ik}C_{jk}\right)-\sum_{k}C_{ik}C_{jk}\right]=\sum_{j}N_{ij}\ln\left(\sum_{k}C_{ik}C_{jk}\right)-\sum_{k}\left(C_{ik}\sum_{j}{R_{ij}C}_{jk}\right)$$

We can get the gradient and Hessian:


(9) $$G_{k}=\partial L_{i}/\partial C_{ik}=\sum_{j}{[N}_{ij}C_{jk}/(\sum_{m}C_{im}C_{jm})-\sum_{j}{R_{ij}C}_{jk}]$$

(10) $$H_{kl}=\partial^{2}L_{i}/\partial C_{ik}\partial C_{il}=-\sum_{j}{[N}_{ij}C_{jk}C_{jl}/{\left(\sum_{m}C_{im}C_{jm}\right)}^{2}]$$

Note that –*H* is a positive-definite matrix, which means that –*L_i_* is a convex function of ***C***_*i*_. Since the constraints $C_{ik}\geq 0$ are also convex, this is a convex optimization problem that can be solved by standard algorithms such as the interior point method. Specifically, we used the primal-dual interior point method with Newton search (Boyd and Vandenberghe 2004) to solve it. By fixing all the ***C***_*j*_ on other chromosomes, all the ***C***_*i*_ on one chromosome can be optimized in parallel.

To optimize α and β, we have the gradients: and the Hessian matrix:


(11) $$G_{\alpha }=\partial L/\partial \alpha =\sum_{ij}\left\{N_{ij}r_{i}r_{j}/[1+\alpha r_{i}r_{j}+\beta (r_{i}+r_{j})r_{i}r_{j}]-r_{i}r_{j}\sum_{k}C_{ik}C_{jk}\right\}$$

(12) $$G_{\beta }=\partial L/\partial \beta =\sum_{ij}\left\{N_{ij}r_{i}r_{j}(r_{i}+r_{j})/[1+\alpha r_{i}r_{j}+\beta (r_{i}+r_{j})r_{i}r_{j}]-r_{i}r_{j}\sum_{k}C_{ik}C_{jk}\right\}$$

(13) $$H_{\alpha \alpha }=\partial^{2}L/\partial \alpha^{2}=-\sum_{ij}\{N_{ij}{\left(r_{i}r_{j}\right)}^{2}/{\left[1+\alpha r_{i}r_{j}+\beta (r_{i}+r_{j})r_{i}r_{j}\right]}^{2}\}$$

(14) $$H_{\beta \beta }=\partial^{2}L/\partial \beta^{2}=-\sum_{ij}\{N_{ij}{\left[r_{i}r_{j}(r_{i}+r_{j})\right]}^{2}/{\left[1+\alpha r_{i}r_{j}+\beta (r_{i}+r_{j})r_{i}r_{j}\right]}^{2}\}$$

(15) $$H_{\alpha \beta }=\partial^{2}L/\partial \alpha \partial \beta =-\sum_{ij}\{N_{ij}{\left(r_{i}r_{j}\right)}^{2}(r_{i}+r_{j})/{\left[1+\alpha r_{i}r_{j}+\beta (r_{i}+r_{j})r_{i}r_{j}\right]}^{2}\}$$

Since –*H* is a positive-definite matrix, –*L* is also a convex function for the vector (α, β) when all the ***C***_*i*_ are fixed, and the optimization problem can be solved by Newton method.

Finally, the whole algorithm works by sequentially optimize ***C***_*i*_ on each chromosome, and then optimize α and β. By iterating this procedure, each time the likelihood function will only increase so the algorithm will finally converge to a local maximum. But since the likelihood function is not overall convex, a global maximum is not guaranteed. When the number of sub-compartments *M* is large, the likelihood landscape is quite complicated, and the algorithm often converges to a local maximum. In this case, we use multiple random starting points and compare the final likelihood value to select the best one.

#### 2.1.3. Excluded regions and region groups

The existing genome assemblies are not perfect, and the real genome may be different among cell lines. Some of the regions have been identified to have abnormal results in genome sequencing experiments and are “blacklisted” (Amemiya et al. 2019). We also excluded these blacklisted regions from our analysis. All blacklisted regions will have all their “***C***_*i*_”s set to 0.

Moreover, there are genome translocations which make *trans*-interactions *cis*, and resulted in much more interactions than expected because genomic regions in the same chromosome interact at much higher frequency. These regions will form a separate compartment in the result because of the high interaction frequency and are easy to identify from normal compartments. The users can also refer to other software or data sources for genomic translocations. When such a translocation compartment is identified, a “blacklisted region group” can be made, and in the algorithm, any pairs of regions within a blacklisted group would not be used in the analysis, which means in the Equation (4), only those region pairs not within the same excluded group will be used.

### 2.2 Additional conditions when the model hypotheses can be violated and the consequences

We have introduced the three hypotheses for the model above. We outline them here and discuss them one by one.

Hypothesis 1: For two different genomic loci *i* and *j*, their nuclear sub-compartment locations in individual cells are independent.

It is easy to imagine that if the two genomic loci are close to each other, like in the same topological-associated domains, the hypothesis will not be satisfied. Therefore, this hypothesis will not hold for all pairs of genomic loci. However, if we only consider *trans*-interactions, the locations of two genomic loci on different chromosomes should be mostly independent.

One possibility we do need to consider is that if the cell population is structurally heterogeneous, i.e. it is a mixture of two or more different cell populations, which could be either different cell types or different cell states, then the independency hypothesis may be violated.

Consider there are two different cell populations, then Equation (3) becomes


(16). $$F_{ij}=R_{ij}\sum_{k}C_{ik}C_{jk}+{R'}_{ij}\sum_{k}{C'}_{ik}{C'}_{jk}$$

In this case, the Equation (3) still maintains its form if the Rabl effects are similar. Only the interpretation of the *C_ik_* values needs to be changed, which means Equations (6) and (7) may not hold. This means we will need a more careful interpretation of the Cscores when dealing with a mixed cell population. On the positive side, this means our method can identify structural heterogeneity in the cell population.

Hypothesis 2: Genomic loci located at different nuclear sub-compartments in one cell do not have 3D interaction in that cell.

In the model, the nuclear sub-compartments correspond to physical locations within the nucleus. Therefore, the frequency of interactions between genomic loci in different sub-compartments should be very low. Only when the two loci are located at the boundary of two neighboring sub-compartments, they should have a low chance of interacting with each other. However, when the interaction frequency is low compared to intra-compartment interactions, this will cause only minor effects.

Hypothesis 3: Genomic loci located at the same sub-compartment in one cell have a constant probability of interaction.

This hypothesis is the most likely to be violated since it is very likely DNA sequences in different nuclear sub-compartments will have different organization and structures, and thus different physical properties and within-compartment interaction frequencies. For example, highly active genomic regions likely have higher *trans*-interaction frequency than condensed heterochromatin. Therefore, our hypothesis has a limitation of over-simplification, and we need to consider the consequences when this hypothesis is violated. Assume for each sub-compartment *k*, the interaction frequency has a modifier *S_k_*, then we have:


(17) $$F_{ij}=B_{i}B_{j}f_{ij}=B_{i}B_{j}R_{ij}\sum_{k}{S_{k}P}_{ik}P_{jk}$$

Let $C_{ik}=B_{i}P_{ik}\sqrt{S_{k}}$, then we still have $F_{ij}=R_{ij}\sum_{k}C_{ik}C_{jk}$. This means the Equation (3) still holds in its original form, only the interpretation of *C_ik_* changes. When the interaction is stronger in one sub-compartment, it will cause globally higher *C_ik_* values in that sub-compartment, while weaker interactions will cause lower *C_ik_* values. This will skew the prediction of *P_ik_* to globally higher/lower probability. The relative higher/lower probabilities of sub-compartment localization between regions should still hold.

### 2.3 Simulated Hi-C data generation

As discussed above, the likelihood function (Equation 4) is complicated and non-convex. Though we can try multiple random starting points, it is uncertain whether the algorithm is robust enough to converge close to the correct optimum values or other sub-optimal local minima. To test the robustness of the method, we performed the following simulation tests.

The first simulation was performed according to our generative model with given model parameters. For each *trans* pair of genomic regions *i* and *j*, the Rabl effect *R_ij_* is first calculated using Equation (5). Then the expected number of interactions between the two regions can be calculated with Equation (2). Finally, the number of observed interactions is generated with Poisson distribution using the expected number of interactions as the parameter. If interactions exist, then the interactions will be output into the simulated Hi-C dataset. After iterating all the *trans* pairs, a full Hi-C dataset is simulated. Then CscoreTool-M is run on the simulated Hi-C dataset to infer compartment scores.

For low-depth dataset simulation, a full Hi-C dataset is first simulated, then a low-depth dataset is generated by down-sampling the full dataset to the lower depth.

For simulation using randomized model parameters, we started from the sub-compartment annotations by Rao et al.’s original Gaussian-HMM annotations. We randomly generated Cscores with different noise levels (NL): For each genomic window, the “annotated” compartment (A1, A2, B1, B2, B3, B4) would take a score of random number from uniform distribution U(1-NL, 1), the other compartments take scores of random numbers from U(0, NL). Note that we also take the B4 compartment into consideration here as it is within Rao’s annotation, so even though only a small number of genomic regions are annotated as B4, all genomic windows will have B4 scores. Then we normalized the Cscores and simulated inter-chromosomal Hi-C dataset to the same depth as the 0.01× GM12878 dataset (about 9M *trans* reads). We did the simulation for different noise levels from 0.05 to 0.5.

For mixed dataset simulation, two Hi-C datasets are first simulated independently according to the two sets of parameters. Then the high-depth dataset between the two was down-sampled to match the depth of the lower one. Finally, the two Hi-C datasets were merged to become one mixed dataset.

### 2.4. AIC calculation

We use the AIC (Akaike Information Criterion) analysis for model selection. AIC is a method for model selection based on the likelihood value and number of parameters and lower AIC values indicate a better model. AIC is calculated with the equation:


AIC=2k-2ln⁡L

Here, *k* is the number of parameters calculated by *k=nM *+* *2, where *M* is the number of sub-compartments, and *n* is the number of non-empty genomic windows. *L* is the estimated maximum value of the likelihood function.

### 2.5. Lamin-B1 cTSA-seq and data processing

cTSA-seq was performed and Illumina sequencing libraries were built as in (Tran et al. 2022). Both cTSA-seq reads and input reads were sequenced with Nextseq 500 Illumina sequencer. Reads were mapped to human genome hg19 using bowtie2 with default parameters. Then for each 100 kb (or 10 kb) genomic window, the number of reads falling into the window was counted, and a relative enrichment was calculated by dividing the number of cTSA-seq reads with the number of input reads. Lamin-B1 cTSA-seq result of GM12878 cell line is available in the GEO database (https://www.ncbi.nlm.nih.gov/geo/) and can be accessed with accession number GSE224282.

## 3 Results

### 3.1 Validation on simulated data

**Figure 1.**
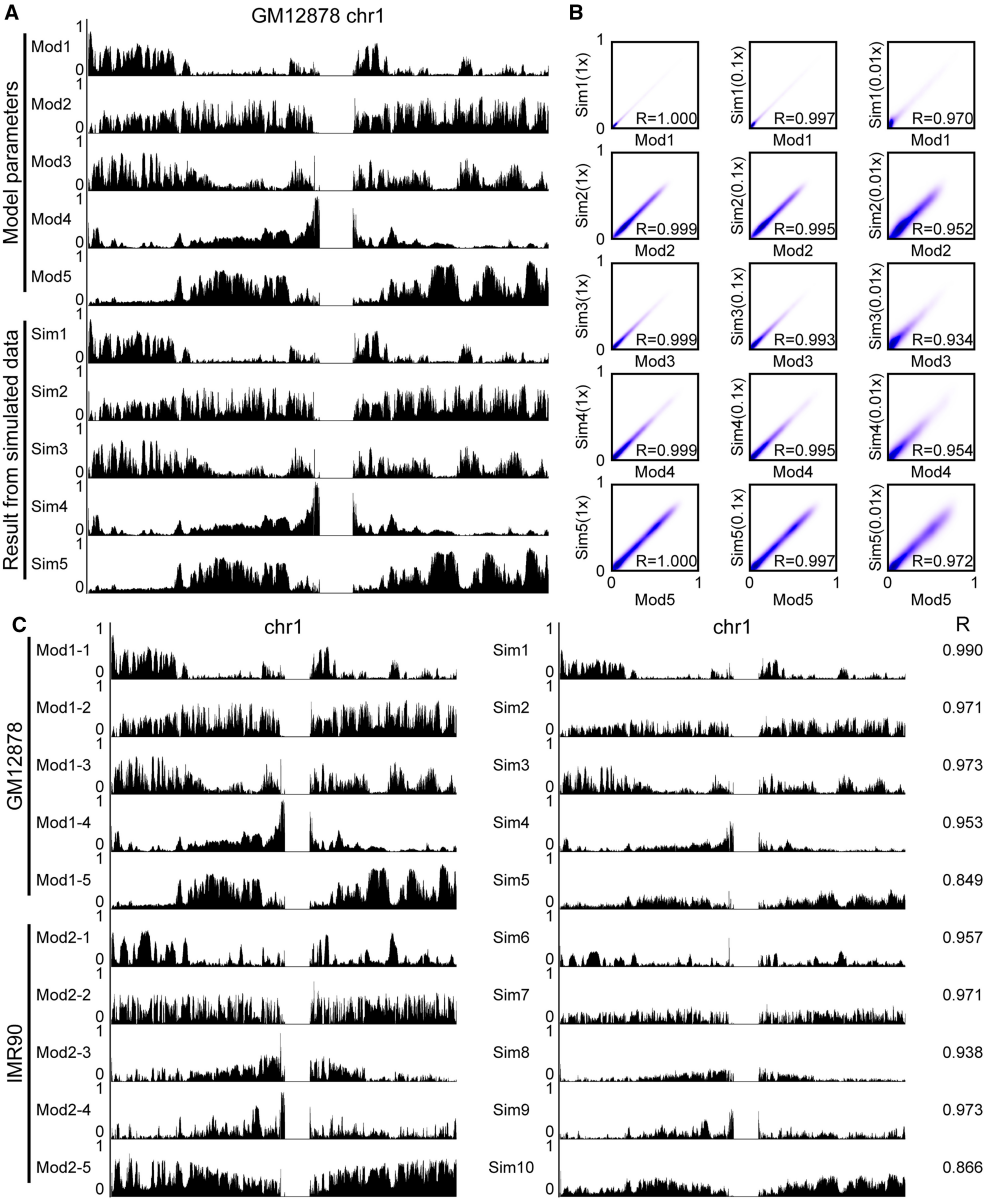
Simulation test using GM12878 cell and IMR90 cell Hi-C datasets. (A) Genome browser view of compartment scores on simulated data (sim1-sim5) compared to the model values (mod1-mod5). Compartment scores on Y axes range from 0 to 1. (B) Contour plots showing the compartment scores calculated on full (1×), low (0.1×), and ultralow (0.01×) depth simulated datasets compared to the model values. R: Pearson correlation coefficient. All X and Y axes compartment score values range from 0 to 1. (C) Genome browser view of compartment scores on simulated mixed dataset compared to the model values. R: Pearson correlation coefficient. All Y axes range from 0 to 1.

To test whether CscoreTool-M can infer the correct sub-compartments, we first performed a simulation test using the GM12878 cell Hi-C data (Rao et al. 2014) as a template. The purpose of this simulation is to test whether the optimization algorithm can correctly estimate the original parameters when all the hypotheses fit. We first ran CscoreTool-M on the GM12878 cell Hi-C data to estimate compartment scores for five sub-compartments at 100 kb resolution using the *trans* interactions (Mod1 to Mod5 on Fig. 1A). Then we simulated Hi-C *trans* interactions at the same depth as the original dataset (1× dataset) using the GM12878 compartment scores as model parameters. We next applied our algorithm on the simulated dataset to infer compartment scores (Sim1 to Sim5 on Fig. 1A).

The chromosomal view of the inferred compartment scores shows high consistency with the model parameters (Fig. 1A). To quantify the divergence between the inferred compartment scores and the model parameters, we calculated the genome-wide correlation coefficients and high correlations are observed for all sub-compartments (Fig. 1B, left panels).

To further test the performance of our method at lower read depth, we generated 0.1× and 0.01× datasets by randomly selecting 10% and 1% from the original dataset, respectively, and calculated the correlation coefficients. The correlations are weaker on low-depth datasets (Fig. 1B, mid and right panels), but they are still higher than 0.9 even on the 0.01× dataset.

We also performed simulation analysis on randomized model parameters using the sub-compartment annotations by Rao et al.’s original Gaussian-HMM as template and randomized to different noise levels. We simulated the Hi-C data, calculated the compartment scores for five compartments and calculated the correlation coefficient with the simulated compartment scores of A1, A2, B1, B2, and B3. We did the analyses for different noise levels from 0.05 to 0.5 and the correlation coefficients between each inferred compartment scores and its corresponding simulated scores are shown in Supplementary Fig. S1. We found that the correlation coefficients become lower when the noise is higher, but all the results are still highly consistent with the simulated scores.

The theoretical analysis (see Section 2.2) suggested that CscoreTool-M can be used to deconvolve mixed cell population. To test this, we simulated two Hi-C datasets using model parameters for GM12878 (Mod1-1 to Mod1-5) and IMR90 Hi-C data (Mod2-1 to Mod2-5) (Fig. 1C). We then mixed these two datasets and applied our algorithm to infer 10 sub-compartments (Sim1 to Sim10 on Fig. 1C). We found five sub-compartments (Sim1 to Sim5) resembled the GM12878 model well (Mod1-1 to Mod1-5), while the other five compartments (Sim6 to Sim10) have a similar profile to the IMR90 model (Mod2-1 to Mod2-5). This result shows that our algorithm can infer cell-type specific sub-compartments in mixed cell types.

### 3.2 Comparisons with the published multi-compartment analyses

To further test the performance of our CscoreTool-M, we compared our results with the published multi-compartments inferred by Rao et al*.* (2014). Rao et al. inferred 6 sub-compartments as A1–A2 and B1–B4 in GM12878 cells. A1 and A2 are both enriched for active chromatin features such as H3K4me3, H3K27Ac, H3K36me3 and DNAse I hypersensitivity. A1 has higher enrichment of active markers than A2 and A1 overlaps with nuclear speckle domains (Chen et al. 2018). B1 to B4 all contain repressed chromatin domains and are depleted of active epigenetic markers. B1 is enriched with H3K27me3 which is regulated by the Polycomb Repressive Complexes. B2 and B3 are both enriched for LADs (determined by the lamin-A/C ChIP-seq), with B2 further enriched of NADs while B3 is not. This suggested that B2 represents NADs while B3 represents LADs. B4 only covers a very small proportion of the genome on chr19. Thus, we excluded B4 and only compared our 5 sub-compartments inferred by CscoreTool-M with the five compartments inferred by Rao et al.

**Figure 2.**
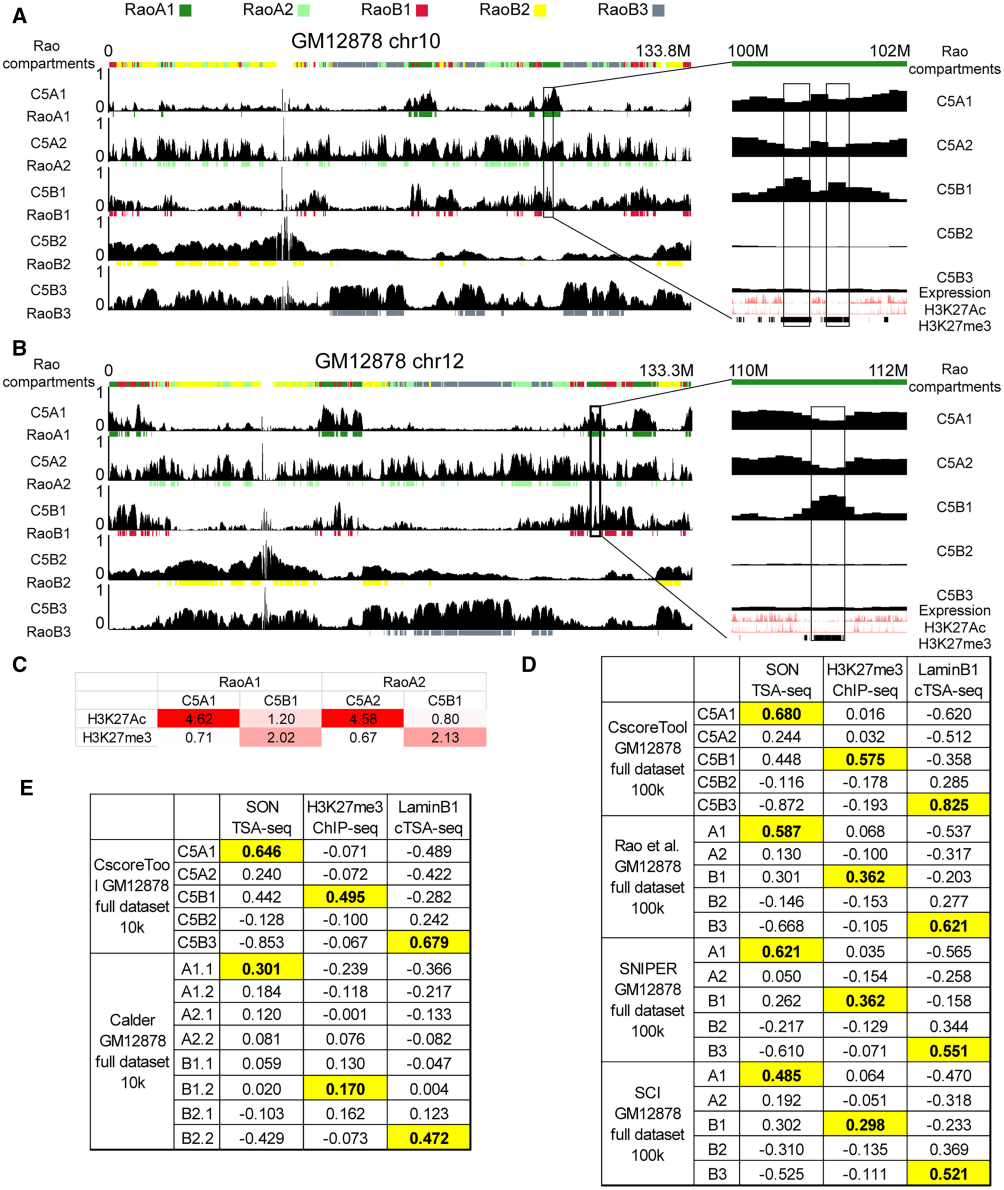
Comparisons of compartments modeled by CscoreTool-M against Rao’s sub compartments in GM12878 cells. (A,B) Genome browser view of chromosome 10 (A) and 12 (B) showing the compartment scores tracks by CscoreTool-M and compartments (indicated by different colored blocks) by Rao et al. Example regions in black rectangles showing discrepancies between CscoreTool-M and Rao et al. are expanded and shown at the right panels. All *Y* axes plot for compartment scores range from 0 to 1. (C) Enrichment of H3K27Ac and H3K27me3 peaks in regions agreed or disagreed inferred by CscoreTool-M or Rao et al. H3K27Ac and H3K27me3 data are from UCSC genome browser ENCODE tracks. (D) Genome-wide correlation coefficients between CsocreTool-M, Rao’s Gaussion-HMM, SNIER, SCI-inferred compartment scores and SON-TSA-seq, H3K27me3 ChIP-seq, Lamin-B1 cTSA-seq enrichment signals at 100-kb resolution. The best scores for each method are highlighted. (E) Genome-wide correlation coefficients between CsocreTool-M, Calder-inferred scores and SON-TSA-seq, H3K27me3 ChIP-seq, Lamin-B1 cTSA-seq enrichment signals at 10-kb resolution. The best scores for each method are highlighted.

We use the same notation as in Rao et al. (RaoA1, RaoA2, RaoB1, RaoB2, and RaoB3) for our five sub-compartments but add “C5” before the compartment names (C5A1, C5A2, C5B1, C5B2, and C5B3) to indicate our CscoreTool-M modeled five sub-compartments. Figure 2A and B shows our five sub-compartment plots based on CscoreTool-M and color-coded bar-tracks for the compartments assigned by Rao et al. In general, our compartment scores show peaks at Rao’s assigned compartments, indicating that our compartment inference is largely consistent with the compartments indicated by Rao. It is noteworthy that there are also regions showing inconsistencies. Figure 2A and B shows that two regions on chr10 and chr12 have C5B1 sub-compartment scores peaks, while Rao et al. predicted these regions to be in A1. To further investigate which sub-compartments these regions should be in, we looked at gene expression and epigenetics features along these regions. Our C5B1 peaks show a depletion of H3K27Ac and reduced gene expression, but enriched for H3K27me3, which are typical B1 features instead of A1.

To further analyse whether the difference in A and B sub-compartment assignment by Rao and by our CscoreTool-M also happens on other chromosomes, we assigned the five compartments based on the highest compartment score at each genomic region across the genome and compared these to the compartments assigned by Rao et al. (Supplementary Fig. S2). While the overall correspondence in A and B sub-compartments is good, there are some clear differences. For example, we found that a total of 45.1M genomic regions are assigned as A1 by Rao but are assigned as C5B1 by CscoreTool-M (Supplementary Fig. S2). We further looked at the enrichment of H3K27Ac peaks and H3K27me3 peaks on these regions (Fig. 2C) and found that the H3K27Ac enrichment (1.2) is just marginally higher than the global average (1.0), while much lower than the enrichment of H3K27Ac peaks on A1 regions assigned commonly by Rao and CscoreTool-M. In contrast, the H3K27me3 peaks showed much higher enrichment (2.02) than that found on A1 regions assigned commonly by Rao and CscoreTool-M (0.71), and also higher than the global average (1.0). We made a similar observation in regions assigned as A2 by Rao and C5B1 by CscoreTool-M. The C5B1 regions show lower H3K27Ac and higher H3K27me3 than the corresponding RaoA2 regions and also than global average (Fig. 2C). These results show that the CscoreTool-M-assigned C5B1 regions have B1 features instead of A1/A2 features. Thus, the CscoreTool-M can better predict sub-compartments in these regions.

To further compare the performance between CscoreTool-M and other published methods, including Rao’s original Gaussian HMM, SNIPER, SCI, and Calder, we used three independent protein-DNA-interaction datasets as benchmarks: SON TSA-seq (of K562 cells) for approximation of A1 (Chen et al. 2018), H3K27me3 ChIP-seq for B1 (Thurman et al. 2012), and lamin-B1 cTSA-seq for B3 (this study). For CscoreTool-M, we used the 5-compartment scores; while for Gaussian-HMM, SNIPER, Calder, and SCI, we used their compartment assignment, 1 for the assigned compartment, and 0 for the others. We calculated the correlation coefficients between the compartment scores generated from each of the sub-compartment analysis methods (CscoreTool-M, Gaussian-HMM, SNIPER, SCI, or Calder) and the enrichment scores generated from each protein-DNA interaction dataset (SON TSA-seq, H3K27me3 ChIP-seq, and Lamin-B1 cTSA-seq). We use these correlation coefficients as a fair comparison as none of Cscoretool-M, Gaussion-HMM, SNIPER, Calder, or SCI directly used this information based on protein-DNA interaction/colocalization datasets. Note that as most results previous are based on the hg19 genome, here the comparisons are also on hg19.

Figure 2D lists the comparison results between CscoreTool-M and Rao’s Gaussian-HMM, SNIPER, and SCI. We found that in all three comparisons for A1, B1, and B3, the results of CscoreTool-M showed the best performance. The comparison against Calder was shown in Fig. 2E. Since Calder provided 10 kb-resolution results, we also applied CscoreTool-M at 10 kb resolution for comparison. In these comparisons, CscoreTool-M also showed better performance than Calder. Taken together, these results show that CscoreTool-M can quantify the sub-compartment probabilities in cell population and the performances are confirmed by other independent measures; and that CscoreTool-M has the best performance in sub-compartment inference among published methods.

We also tested the performance of our CscoreTool-M on low-depth Hi-C sequencing data by randomly selecting 0.1× and 0.01× of the reads from the Hi-C data for GM12878 cells to infer sub-compartments. We then calculated the correlation coefficient on the sub-compartments. We found all the correlation coefficients are higher than 0.9 (Supplementary Fig. S3), indicating that the CscoreTool-M is robust in inferring multiple genomic compartments using low-depth Hi-C datasets. Comparison between biological replicates of GM12878 Hi-C data also showed high correlation (all > 0.97, Supplementary Fig. S4), further supporting the robustness of the method.

One important hyper-parameter of CscoreTool-M and other sub-compartment inference tools is the number of sub-compartments. We first compared 2- and 5-compartment results to see how this hyper-parameter can determine the sub-compartment results. We calculated the correlation coefficients between 2- and 5-compartment scores (Supplementary Fig. S5). A1 has the highest correlation with A compartment score, followed by A2; B3 has the highest correlation with B compartment score, followed by B2. Interestingly, B1 has mildly positive correlation with A compartment score instead of B. It has been reported that the B1 sub-compartment is located farther away from the nuclear periphery than A2 (Robson et al. 2017), indicating that when only considering 2-compartments, it is correct to assign the B1 compartment as mostly A-like. However, with 5-sub-compartment analysis, it is possible to further separate the B1 sub-compartment from the A-compartment and correctly identify its polycomb-repressed features. This result shows that with more sub-compartments, it is possible to identify more detailed information of 3D genome organization.

Since CscoreTool-M uses maximum-likelihood inference, it is natural to use the AIC (Akaike Information Criterion) for model selection. AIC is a method for model selection based on the likelihood value and number of parameters. The lower AIC values indicate a better model. We applied AIC to two datasets (0.1× and 0.01× data of GM12878) at 100 kb resolution. For lower-depth dataset (0.01×), the AIC reached a minimum at 6 compartments, indicating that 6 is the statistical optimal compartment number for this dataset (Supplementary Fig. S6A), while for a higher-depth dataset (0.1×), the AIC values decrease as the number of compartments increase up to 15 compartments (Supplementary Fig. S6B) indicating that more compartments continuously lead to better fitting of the GM12878 Hi-C data at this depth. In this case, the statistical optimal compartment number is at least 15. The users can perform a similar analysis to determine the statistically optimal number of sub-compartments. However, depending on the biological questions and when the sequencing depth is high, the analysis may not need to reach the optimal minimum. In all the bulk-Hi-C datasets we use below, the AIC analysis supports more than 15 sub-compartments, and we used 5, 6, or 10 just because they are enough to support our biological findings.

### 3.3 Inferring LAD and NAD heterogeneity within a cell population

One feature of CscoreTool-M is that it explicitly models and quantifies the heterogeneity within a cell population, which should aid the study of variations of genome organization. It is known that NADs often overlap with LADs, but the overlap only happens for some NADs. The NAD-only and the overlapping NAD/LAD chromatin regions tend to have different genomic features (Vertii et al. 2019; Bersaglieri et al. 2022). Since nucleoli are often not located at the nuclear periphery, the biological interpretation of the overlap between NADs and LADs remains unclear. The localization of lamins in nucleolus as reported by some studies (Martin et al. 2009; Sen Gupta 2017) can contribute to the NAD/LAD overlap. In addition, only a fraction of LADs may return to the nuclear lamina after mitosis, and the ones not returning to the nuclear lamina often relocate to peri-nucleolar regions (Kind et al. 2013). In this case, the overlapping LADs and NADs regions can come from heterogeneity within the cell population, with the same regions located at the nucleoli or nuclear periphery in different cells within the population.

**Figure 3.**
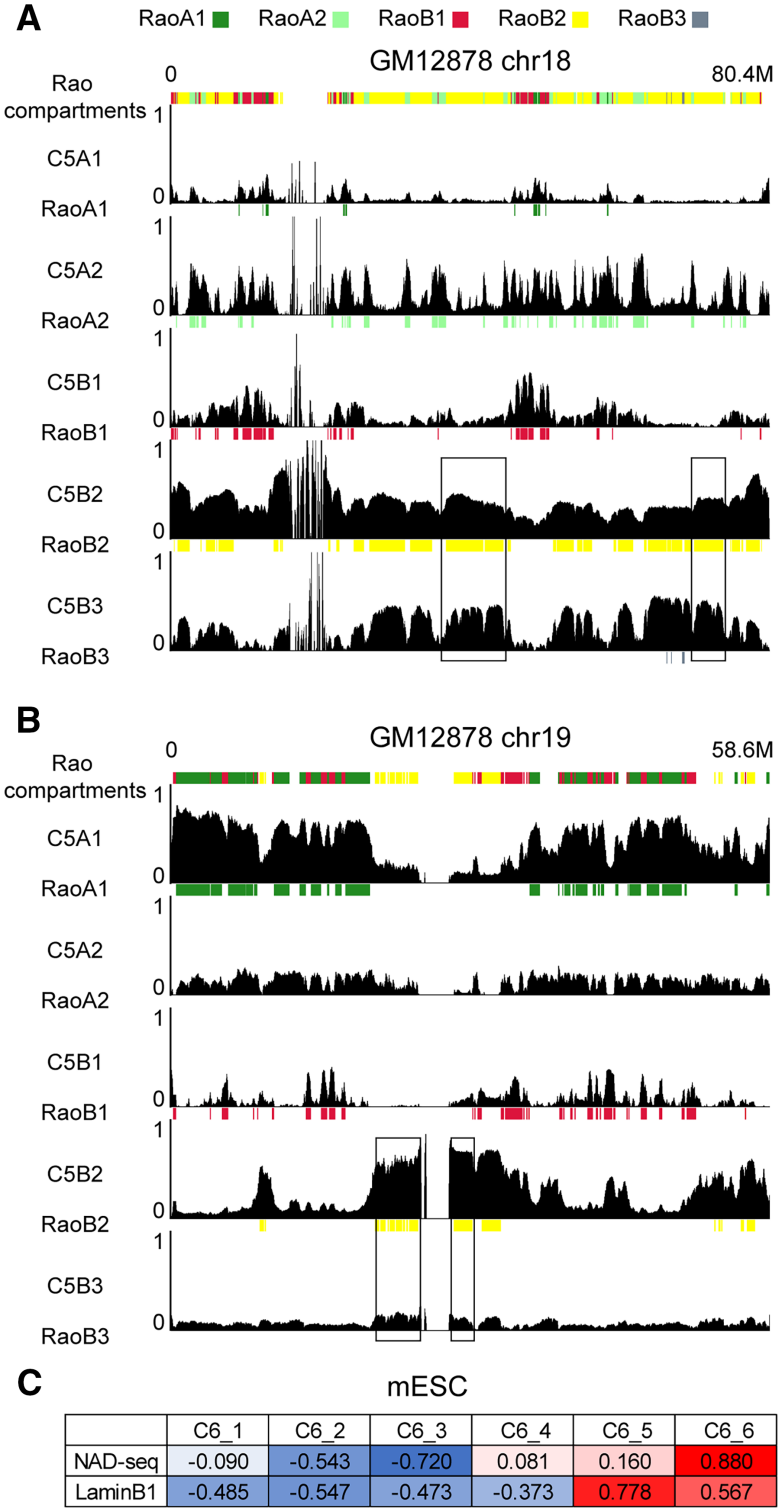
Inferring NADs and LADs in GM12878 cells. Genome browser view of chromosome 18 (A) and 19 (B) showing the CscoreTool-M compartment score tracks and the corresponding compartments by inferred by Rao et al. Black rectangles indicate two examples of RaoB2 regions showing high C5B2 and C5B3 scores on chr18 (A) or two examples of RaoB2 regions with high C5B2 and low C5B3 scores on chr19 (B). All Y axes plotting compartment scores range from 0 to 1. (C) Genome-wide correlation coefficients between CscoreTool-M inferred 6-subcompartment scores and NAD-seq, Lamin-B1 DamID enrichment signals at 100-kb resolution.

We applied CscoreTool-M to infer LADs and NADs based on the Hi-C dataset for the GM12878 cell population. One interesting feature we noticed is that our compartment scores show clear variations among Rao et al’s B2 chromatin regions. Examples are shown for chr18 and chr19 (Fig. 3A and B), which are known to localize preferentially to the nuclear periphery and nuclear interior, respectively (Croft et al. 1999). On chr18, about half of the chromosome was assigned as B2 by Rao et al. (RaoB2), but based on Cscoretool-M these regions have similar C5B2 and C5B3 scores. Although RaoB2 and RaoB3 are suggested to represent NADs and LADs regions, respectively, our analyses indicate that the RaoB2 chromatin regions on chr18 exhibit cell-cell heterogeneity with about half of the cells having nucleolar localization and the other half localizing at the nuclear lamina. In contrast to chr18, CscoreTool-M found that the RaoB2 regions on chr19 have high C5B2 scores and low C5B3 scores, suggesting that these regions are NADs but not LADs. This correctly infers that the interiorly localized chr19 has limited LADs compared to that of chr18.

Since there is no NADs data on GM12878 or similar cell types, we tested the performance of CscoreTool-M to quantify the heterogeneity of LADs and NADs on mouse embryonic stem cells (mESC). We downloaded NAD-seq data from (Bizhanova et al. 2020), Lamin-B1 DamID data from (Peric-Hupkes et al. 2010), and calculated NAD-seq and Lamin-B1 DamID enrichment signals on 100 kb windows along the genome. We then applied CscoreTool-M to mESC Hi-C data (Bonev et al. 2017), and calculated the correlation coefficients with NAD-seq and Lamin-B1 DamID (Fig. 3C). We found that with six sub-compartments, one of the sub-compartments, C6_6, has a very high correlation coefficient (0.88) with NAD-seq, and only 0.567 with LaminB1 DamID. In contrast, another sub-compartment, C6_5, has a high correlation coefficient (0.778) with LaminB1 DamID, while only 0.160 with NAD-seq. This result shows that CscoreTool-M can successfully identify separate sub-compartments that correspond to NADs and LADs, respectively. The high correlation coefficients against NAD-seq and Lamin-B1 DamID signals further confirm that the probabilities of locating in LADs or NADs inferred by CscoreTool-M are highly consistent with the results from independent measurements. Taken together, our method can infer the heterogeneity of NADs and LADs localization and quantify the different levels of heterogeneity among genomic regions within the cell population.

### 3.4 Inferring the degree of compartment heterogeneity in different cell types

**Figure 4.**
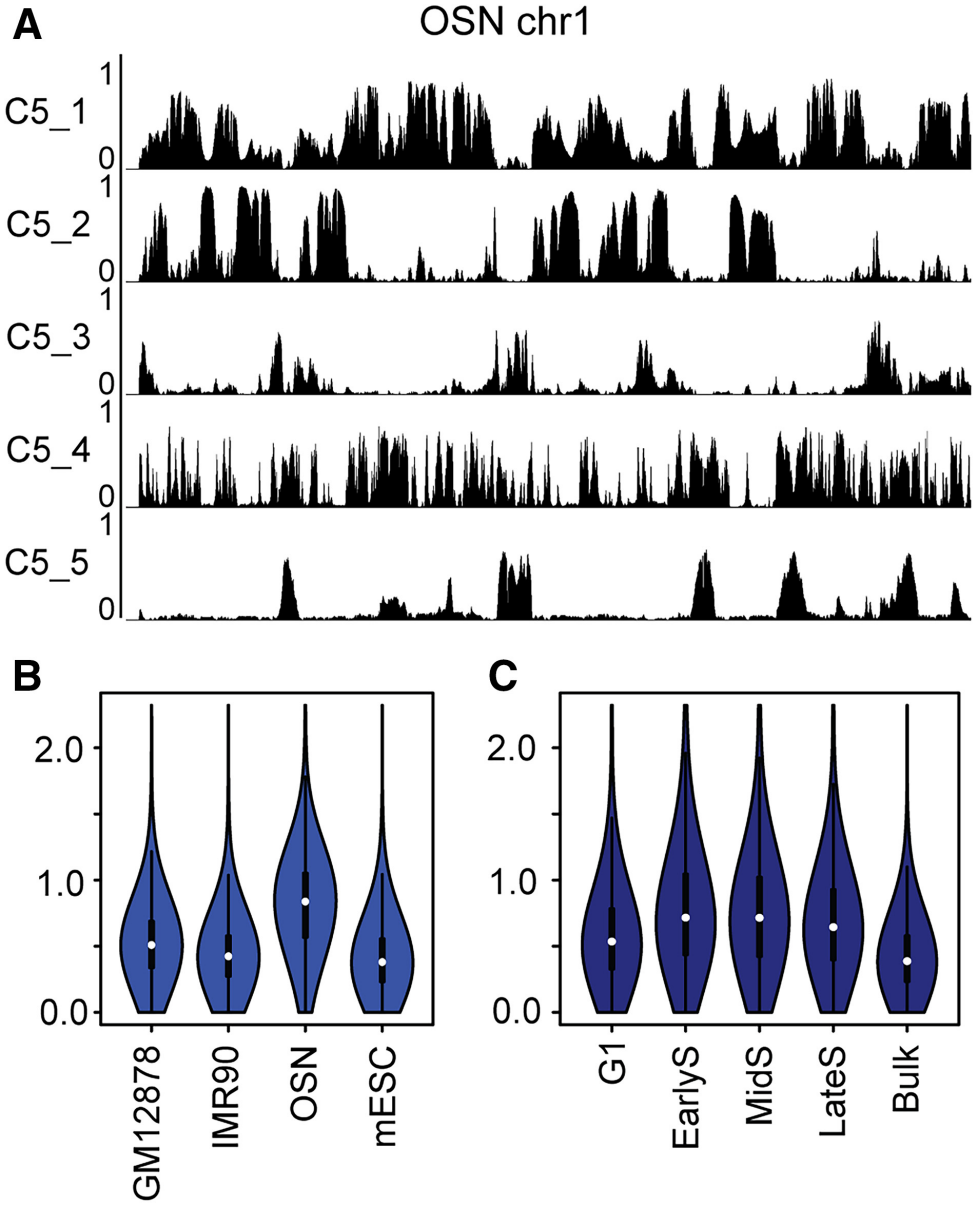
Comparisons of the level of compartment heterogeneity among different cell types. (A) Genome browser view of five compartments for olfactory sensory neurons (OSN) on chr1 inferred by CscoreTool-M. All *Y* axes plotting compartment scores range from 0 to 1. (B) Violin plot showing the information content calculated for each 100-kb window along the genome based on Hi-C datasets in four different cell types. (C) Violin plot showing the information content calculated for each 100-kb window along the genome based on single-cell Hi-C datasets in four cell-cycle stages of mESC compared to bulk.

Studies have revealed clear variations in 3D genome interactions in individual cells belonging to the same cell type. These variations can be purely stochastic or related to different cell states, such as different cell-cycle or differentiation stages. On the other hand, a given cell type, i.e. not proliferating and is terminally differentiated should exhibit relatively low heterogeneity in genome organization. Our CscoreTool-M should be able to infer the degree of 3D genome organizational heterogeneities among different cell types and within a population of cells belonging to the same cell type or lineage. To test this, we applied CscoreTool-M to analyse the Hi-C dataset for the isolated pure mature olfactory sensory neurons (OSN) (Monahan et al. 2019). The result of the 5-compartment analyses (Fig. 4A) shows sharper sub-compartment boundaries and lower background scores than those of the five compartments inferred based on the Hi-C datasets for GM12878 and IMR90 cells (see Fig. 1C).

To further quantify the degree of heterogeneity, we calculated the information content (IC) of sub-compartment scores along the genome. IC is defined as $I=\sum_{i}\left[p_{i}\log_{2}{(p}_{i}/0.2)\right]$ for 5 sub-compartments, is highest if the sub-compartment score is 1 for one sub-compartment and 0 for the remaining compartments (no heterogeneity), while lowest (0) if it is the same for all sub-compartments (no information). We found that IC for the olfactory neuron Hi-C dataset is generally higher than the GM12878 and IMR90 Hi-C datasets (Fig. 4B), confirming our visual inspection of the genomic tracks. Since the olfactory sensory neurons were isolated from mice, we compared the information content of the mouse olfactory neuron Hi-C with a mouse ES cell (mESC) Hi-C dataset (Bonev et al. 2017). The olfactory neuron dataset still has significantly higher IC (Fig. 4B). Thus, the difference is not due to the differences of cells derived from mice or humans.

The above analysis suggests that cells that continue to proliferate may result in increased genome organization heterogeneity, and that cell cycle and the related chromatin conformation change is one major reason for heterogeneity. To test this hypothesis further directly, we applied CscoreTool-M to five cell-cycle-stage-sorted single-cell Hi-C datasets (Nagano et al. 2017) corresponding to G1, early S, mid S and late S/G2, respectively. We then applied 5-compartment analysis to these cell-cycle-resolved datasets and calculated the IC in all four cell cycle stages. We found that the IC values were higher than in the bulk mESC Hi-C data (Fig. 4C). We also found that the IC values were lower in G1 than in early S, mid S, and late S/G2, consistent with the idea that the G1 phase is the most heterogeneous with rapid chromatin interaction changes from the mitotic to interphase conformation. These results show that CscoreTool-M can be used to infer and compare genomic organizational heterogeneity in different cell types, and that cell cycle is a major reason for sub-compartment heterogeneity.

### 3.5 Inferring G1 genome organization in a population of asynchronously dividing cells

**Figure 5.**
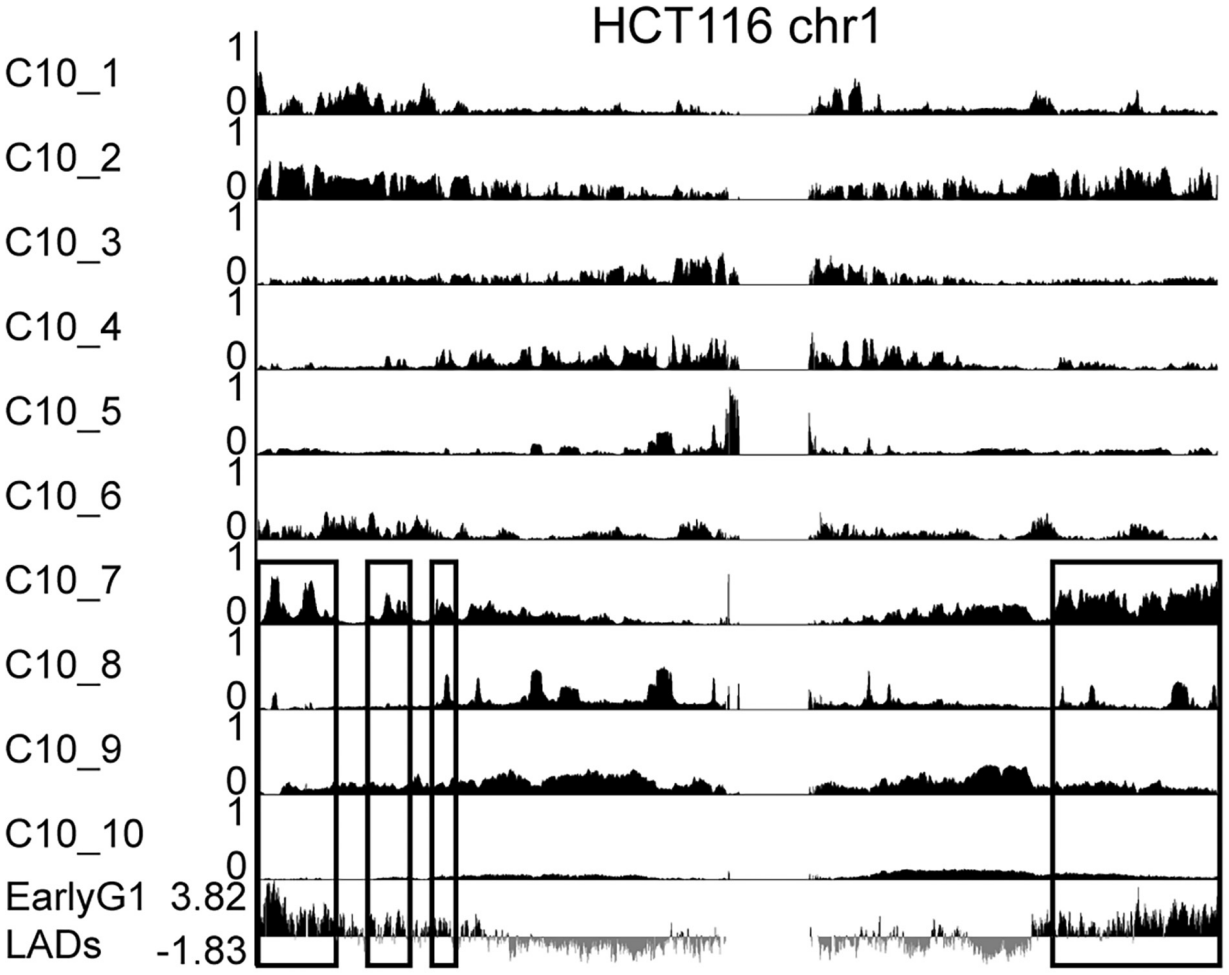
Inferring cell-cycle-related sub-compartments. Genome browser view of 10 compartments inferred by CscoreTool-M for HCT116 cells on chr1. Regions showing similarity between HCT116 C10_7 compartment and the early-G1-specific LADs determined by cTSA-seq using sorted early G1 HCT116 cells are highlighted in black boxes.

In a given cell type, those cells that are proliferating can have different 3D genome interactions at different cell cycle stages, thereby contributing to the variation in chromatin organization among individual cells. Our simulation analysis suggests that the CscoreTool-M can deconvolve genomic organizational heterogeneity if we analyse a larger number of sub-compartments. We analyzed the Hi-C dataset obtained from the asynchronously dividing HCT116 cells (Rao et al. 2017) using 10 sub-compartments (Fig. 5). Recently we have developed a chromatin pull-down based Tyramide-Signal Amplification sequencing (cTSA-seq) method to analyse LADs at different cell cycle stages using the human HCT116 cell line (Tran et al. 2022). We found that during early G1 the chromatin associated with the reforming NL are the sub-telomeric regions (Fig. 5). Another study using a novel protein A-DamID approach described a similar, albeit weaker telomeric enrichment (van Schaik et al. 2020). Interestingly, one sub-compartment, HCT116 C10_7, predicted by CscoreTool-M is very similar to the early G1 LADs mapped by our cTSA-seq. The whole-genome comparison shows significant correlation between the compartment score of HCT116 C10_7 and the early G1 cTSA-seq LADs mapping (*R* = 0.42, *P* < 10^−16^). This result shows that the C10_7 sub-compartment can capture the LADs pattern in the early G1 HCT116 cells.

### 3.6 Inferring cell-type specific functional sub-compartments

**Figure 6.**
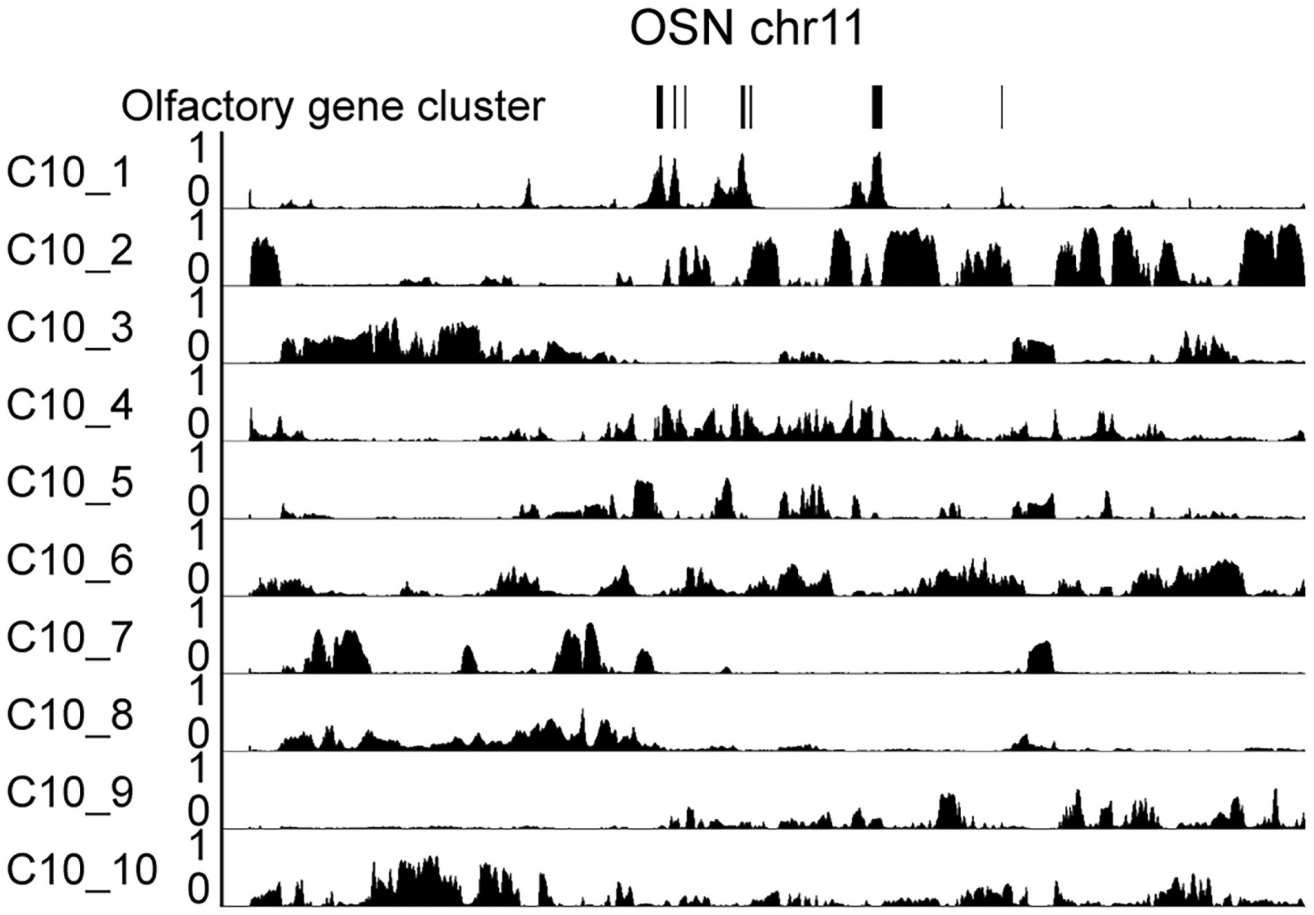
Identification of function-related sub-compartments in OSN by 10 sub-compartment analyses. Genome browser view of 10-compartments for olfactory sensory neuron cells on chr11. The olfactory gene clusters are indicated with black bars on the top. Thickness of the bars indicate the number of olfactory genes.

One interesting feature of the olfactory neuron is that the olfactory genes are clustered together in 3D (Monahan et al. 2019). To test whether our method can find this sub-compartment of olfactory genes, we applied the CscoreTool-M on the olfactory sensory neuron Hi-C dataset to model 10 sub-compartments. We found that one of the sub-compartments, OSN C10_1, overlapped well with the olfactory genes (Fig. 6), indicating that our method can identify this functional gene cluster in one sub-compartment.

We reason that these functional gene clusters may also exist in other cell types. We first examined the GM12878 dataset. We performed 10-compartment analysis on the GM12878 dataset and found that the GM12878 C10_1 and C10_2 sub-compartments both have good correlations to the GM12878 C5A1 compartment but are enriched for genes with different functions based on the Gene Ontology (GO) analyses. The C10_1 sub-compartment is enriched for genes related to immune function, which may be functionally important as the GM12878 cells are derived from B-lymphocytes (Supplementary Fig. S7A). In contrast, the C10_2 sub-compartment is enriched for housekeeping genes with functions including translation and transcription (Supplementary Fig. S7B). Interestingly, we found that the C10_2 sub-compartment for the olfactory sensory neurons is also enriched for housekeeping genes (Supplementary Fig. S7C). Finally, we applied CscoreTool-M to infer 10 sub-compartments in additional cell types with available Hi-C datasets to test if we can find 3D clustering of functionally related genes in individual genomic compartments. By analyzing mESCs, IMR90 fibroblasts, NHEK keratinocytes, and HCT116 colon cancer epithelial cells, we found a clear enrichment of housekeeping genes in two sub-compartments of mESC (Supplementary Fig. S8A and B), one in IMR90 (Supplementary Fig. S8D) and one in HCT116 cells (Supplementary Fig. S8E). GO analyses also revealed an enrichment of genes in the C10_3 compartment of mESC involved in embryonic development (Supplementary Fig. S8C) and genes in the C10_2 compartment of NHEK cells involved in keratinocyte differentiation and skin development (Supplementary Fig. S8F). Thus, CscoreTool-M can discover the 3D cluster of functionally related genes in different cell types.

## 4 Conclusions

In this study, we report CscoreTool-M, a model-based method to calculate sub-compartment scores from Hi-C data. The compartment scores calculated by Cscoretool-M for a given genomic region are directly proportional to the probability that the region is located at a specific sub-compartment. By comparing with other sub-compartment inference based on clustering method, we have shown that CscoreTool-M is better at inferring sub-compartments corresponding to both active and repressed chromatin. The compartment scores by CscoreTool-M also help quantify the levels of heterogeneity in sub-compartment localization within cell populations for different genomic regions. By comparing proliferating and terminally differentiated cells, we show that proliferating cells have higher genome organization heterogeneity, which is likely caused by different cell-cycle stages. By analyzing 10 sub-compartments, we found a sub-compartment potentially related to early-G1 LADs in HCT116 cells, suggesting the method can deconvolve sub-compartments from asynchronously dividing cell populations. Finally, we show that sub-compartments inferred by CscoreTool-M are often enriched in housekeeping or cell-type-specific functions.

## Supplementary Material

btad314_Supplementary_Data
