# Supplementary material for: CscoreTool-M infers 3D sub-compartment probabilities within cell population
Source: Bioinformatics. 2023 May 11;39(5):btad314. doi: 10.1093/bioinformatics/btad314 (PMC10206090; doi:10.1093/bioinformatics/btad314)
Supplement: btad314_Supplementary_Data [file btad314_supplementary_data.docx]

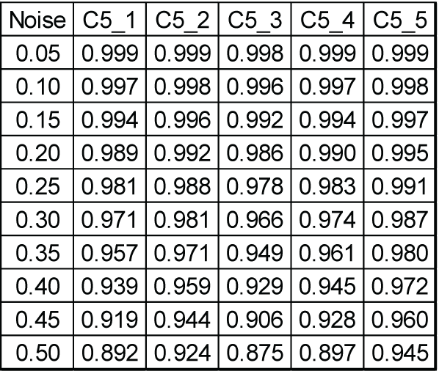


Fig. S1. Correlation coefficients between compartment scores inferred from simulated Hi-C data and the corresponding model parameters at different noise levels.


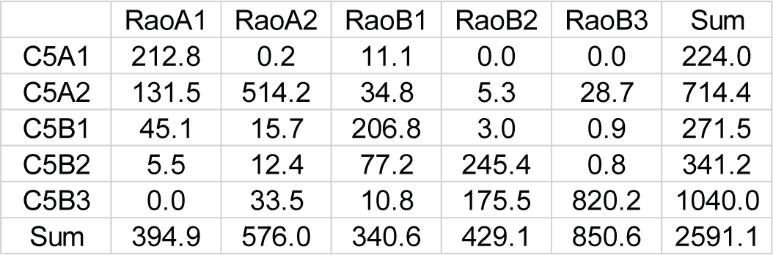


Fig. S2 Comparisons between compartments inferred by CscoreTool-M and Rao et al. Values are in million bases.


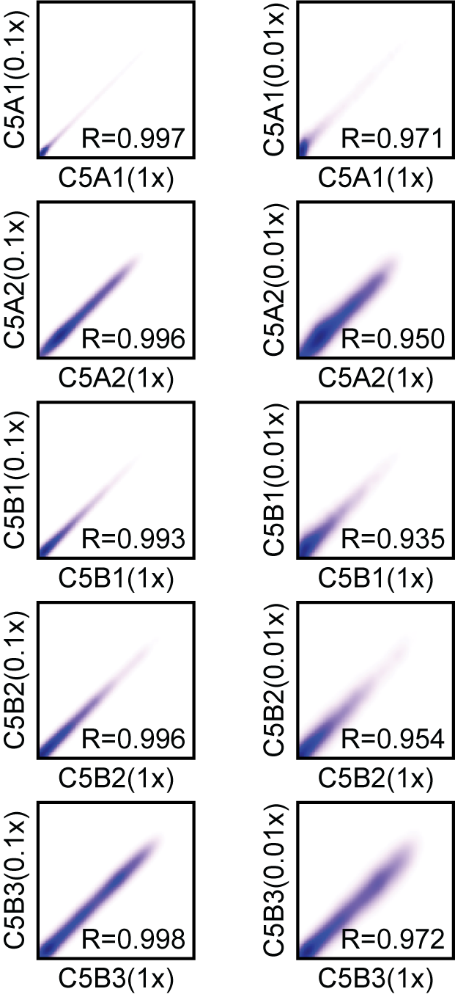


Fig. S3 Contour plots showing the compartment scores calculated on low (0.1x) and ultralow (0.01x) depth datasets compared to the original dataset. All compartment score values on X and Y axes range from 0 to 1.


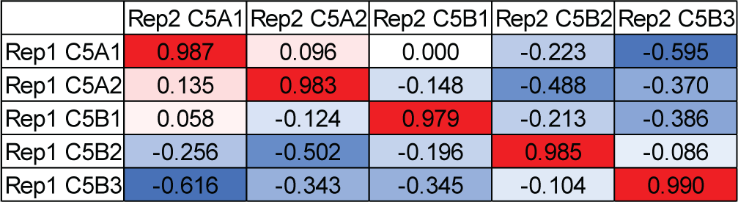


Fig. S4. Correlation coefficients between sub-compartment scores inferred from two replicates of GM12878 Hi-C data.


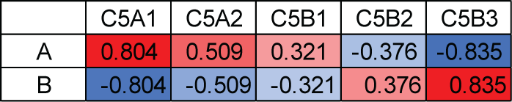


Fig. S5 Correlation coefficients between 2-compartment and 5-subcompartment scores.


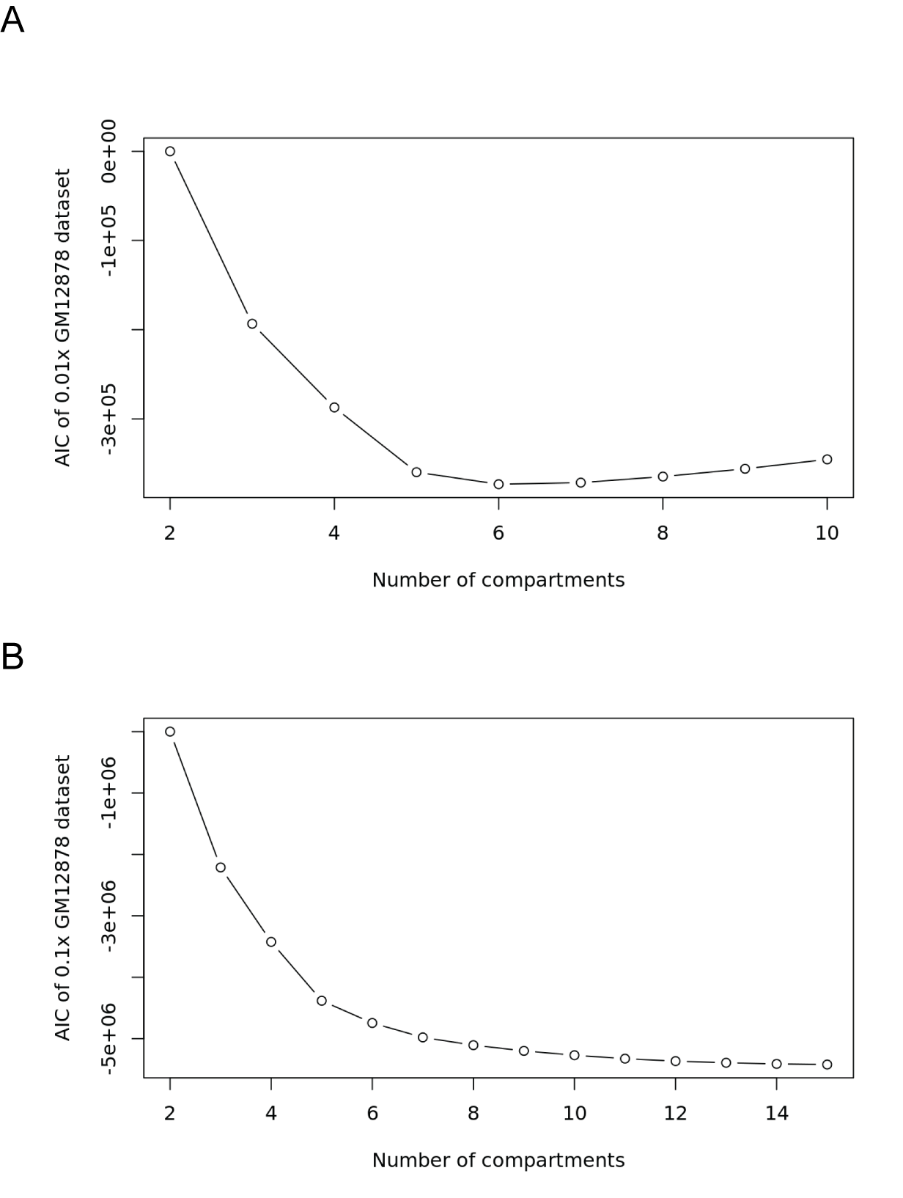


Fig. S6 AIC analysis results of 0.01x (A) and 0.1x (B) GM12878 Hi-C datasets. Note the AIC values are relative to the two-compartment values.


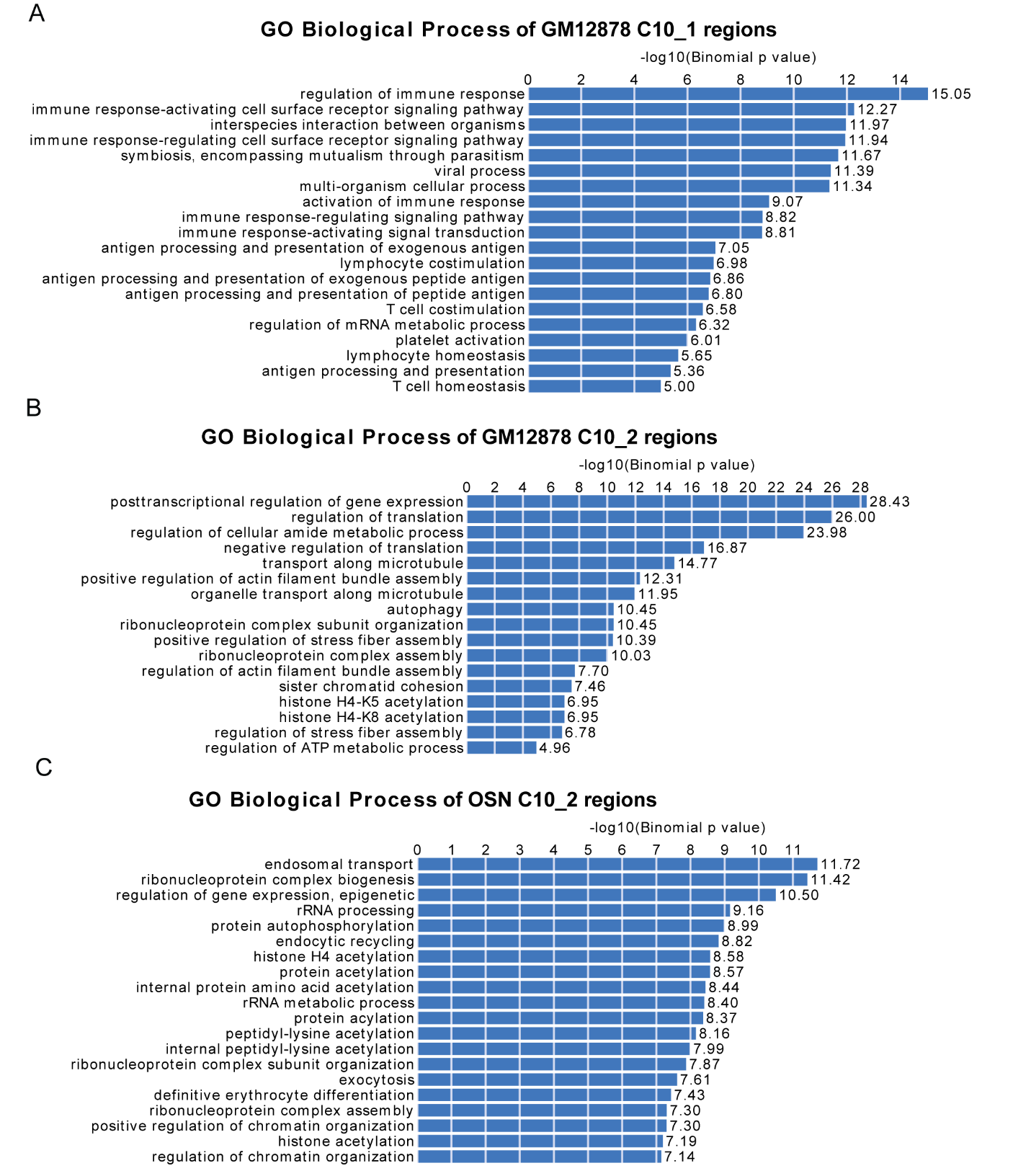


Fig. S7 Identification of function-related sub-compartments in GM12878 and OSN by 10 sub-compartment analyses. A-B. GO-term enrichments by GREAT of genes in the compartment C10_1 (A) and C10_2 (B) compartments in GM12878 cell. C. GO-term enrichments of the C10_2 compartment in olfactory and sensory neurons (OSN).


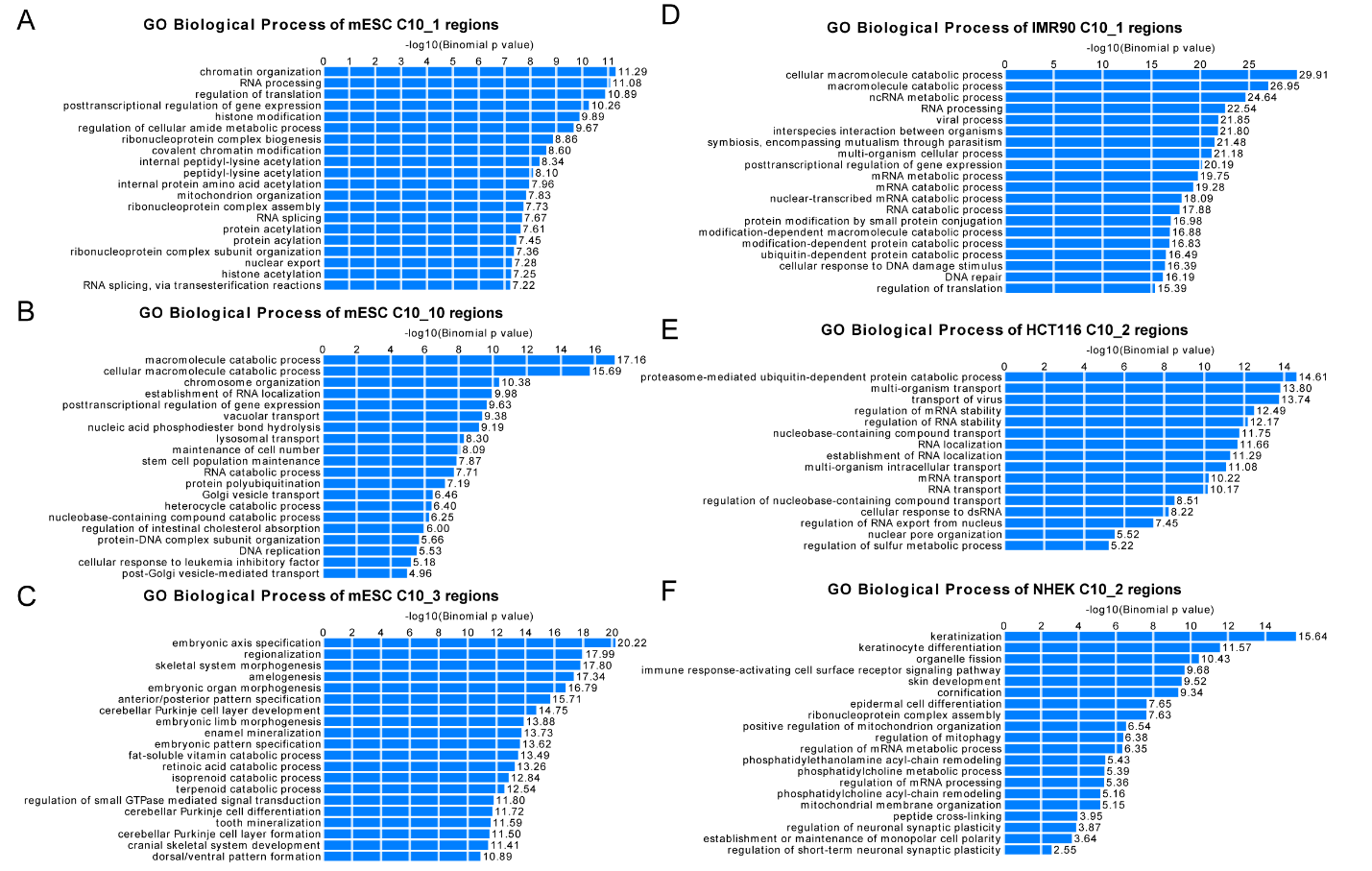


Fig. S8 GO-term analysis of functional-related sub-compartments based on 10 sub-compartment modeling in mESCs (A-C), IMR90 (D), HCT116 (E) and NHEK(F) cells.
